# Radionuclides: medicinal products or rather starting materials?

**DOI:** 10.1186/s41181-019-0074-3

**Published:** 2019-08-20

**Authors:** Oliver Neels, Marianne Patt, Clemens Decristoforo

**Affiliations:** 1 Division of Radiopharmaceutical Chemistry, German Cancer Research Center (dkfz), 69120 Heidelberg, Germany; 2 Department for Nuclear Medicine, Radiochemistry, University of Leipzig, 04103 Leipzig, Germany; 3 Department of Nuclear Medicine, Medical University Innsbruck, 6020 Innsbruck, Austria

**Keywords:** Radionuclides, Regulatory, Medicinal product

## Abstract

The EU directive 2001/83 describes the community code for medicinal products for human use including radiopharmaceuticals. In its current definition, also radionuclide precursors, such as fluorine-18, need to hold a marketing authorization before being placed on the market. The potential of novel radiopharmaceuticals for nuclear medicine is, although encouraged by European legislation and its respective guidance documents, therefore hampered by the regulatory framework. An update of EU directive 2001/83 would be beneficial for the development of novel radiopharmaceuticals and a safe advance in nuclear medicine.

## Background

Nuclear Medicine has shown its high potential in personalized medicine and targeted therapy approaches, in particular increasingly implementing PET into clinical routine and establishing novel targeted therapies in oncology (Fanti et al. 2018). It thereby takes advantage of the versatility of preparing radiopharmaceuticals on-site, starting with a radionuclide with a short half-life of minutes to a few days. The radionuclide is either produced in a reactor, by a cyclotron or obtained by elution of a radionuclide generator. While the use of a radionuclide generator as the source of the radionuclides is relatively easy to realize, the radionuclide production in a reactor is restricted to very few sites that can manage the technically demanding requirements. The use of cyclotron produced radionuclides requires either a cyclotron on-site or the need to ship the radionuclides from external suppliers.

Currently within the European Union (EU) pharmaceuticals are covered within directive 2001/83 (The European Parliament and the Council of the European Union 2001). It defines radiopharmaceuticals as “*Any medicinal product which, when ready for use, contains one or more radionuclides (radioactive isotopes) included for a medicinal purpose.*”

It has to be clearly stated, that the use of novel radiotracers, that are currently accepted as “established” radiotracers, is in principle possible and rightfully encouraged by European legislation and respective guidance documents (EDQM 2019; PIC/S Guide to Good Practices for the Preparation of Medicinal Products in Healthcare Establishments PE 010-4 2014; The European Parliament and the Council of the European Union 2014). Regulatory bodies in Europe rightfully acknowledged, that new diagnostic tools (especially within the field of PET-radiopharmaceuticals) should be available faster than through the tedious way of getting a new diagnostic tool authorized via EMA (or the national authorities). However, the good and justified intent of the legislative bodies is severely hampered by the more general legislation on radiopharmaceuticals (The European Parliament and the Council of the European Union 2001).

Within the EU, medicinal products have to be licensed, i.e. have to hold a marketing authorization when placed on the market. As a reasonable consequence this applies as well for radiopharmaceuticals unless they are prepared within a hospital for internal/local use. But in addition to that it also is applicable to radionuclide generators, (non-radioactive) kits and so-called radionuclide precursors, the latter ones being defined by the afore mentioned directive as “*Any other radionuclide produced for the radio-labelling of another substance prior to administration*”. Therefore, a clinical institution (for example a University Hospital), well equipped with a cGMP compliant radiopharmacy, but without a cyclotron on-site, is unable to prepare any ^18^F-labelled radiotracers for patient use simply because they cannot legally purchase one of the required starting materials, i.e. the radionuclide precursor “^18^F fluoride for radiolabelling”.

In our view, there is no need to treat radionuclide precursors, such as fluorine-18, different from other starting materials. Starting materials for the preparation of pharmaceuticals according to current legislation have to be controlled by

- performing several tests on the starting material itself (identity, purity, assay, etc.).
- documentation of all necessary quality aspects.
- assurance of a suitable quality management system at the suppliers site.

A similar situation is evolving in radionuclide therapy applications. A number of radionuclides, in particular alpha emitters, have shown highly encouraging clinical utility (Poty et al. 2018). Several of the required production routes, that are at this moment not covered by pharmaceutical producers, are provided by highly specialized research institutions using technically challenging techniques of radionuclide separation and receiving considerable funding for developing novel radionuclides for medical purposes by public bodies, in particular the European Commission.

We propose, to reconsider the regulatory requirements for radionuclide precursors; i.e. instead of defining them as medicinal products, radionuclide precursors should be treated as a starting material, unless they are used directly in combination with licenced kits. As for all other starting materials used in the production of medicinal products, the manufacturer of the final radiopharmaceutical has to ensure that all starting materials, including the radionuclide precursor, are of appropriate quality suitable for the intended preparation process.

The current definition of radionuclide precursors, generators and kits was introduced considering the common practice of using radionuclides in combination with kits (e.g. [^111^In]InCl_3_ in Octreoscan®, [^90^Y]YCl_3_ in combination with Zevalin®). The final radiopharmaceutical is prepared in a very simple process and only very simplified quality control procedures are applied. If this practice is more clearly linked to the current definition and not enforced onto radionuclides introduced in a more complex, much more strictly and densely controlled way, such as in the case of fluorine-18, it would ensure the access to novel radiopharmaceuticals without impairing the quality and safety for the patient. The definition of a radionuclide precursor should be changed to “*Any other radionuclide produced for the direct radio-labelling of another substance prior to administration without further processing*”. The respective guidelines (European Medicines Agency 2019) should be adapted to clarify and distinguish between the direct use of a radionuclide without appropriate validation of synthesis processes and final testing as compared to the situation where the radionuclide is introduced in a well-controlled process with final testing of the radiopharmaceutical according to e.g. the European Pharmacopoeia standards. Thereby it should be independent of the source of the radionuclide, being a cyclotron, generator or reactor.

## Conclusion

We believe, that the great potential of novel radiopharmaceuticals for Nuclear Medicine can only be fully exploited, if the availability of a growing number of clinical radionuclides is ensured and not hampered by the regulatory framework. We have described an urgent need for novel developments in radiopharmaceutical sciences to be followed-up by the regulatory framework. Avoiding unnecessary cost and delays for implementation of radiopharmaceuticals will be of great benefit for both patient and healthcare in general. Updating EU directive 2001/83 will help the safe advance in nuclear medicine and radiopharmaceutical product developments.

## Data Availability

Not applicable.

## Abbreviations

cGMP: Current Good Manufacturing Practice
EMA: European Medicines Agency
EU: European Union
PET: Positron Emission Tomography
